# Traumatic Brain Injury, Sleep, and Melatonin—Intrinsic Changes with Therapeutic Potential

**DOI:** 10.3390/clockssleep5020016

**Published:** 2023-04-06

**Authors:** Allen Bell, Bryson Hewins, Courtney Bishop, Amanda Fortin, Jonathan Wang, Jennifer L. Creamer, Jacob Collen, J. Kent Werner

**Affiliations:** 1 Walter Reed National Military Medical Center, Bethesda, MD 20814, USA; 2 School of Medicine, Uniformed Services University of the Health Sciences, Bethesda, MD 20814, USA; bryson.hewins@usuhs.edu (B.H.);

**Keywords:** traumatic brain injury, melatonin, insomnia, therapeutic, neurology, neuroinflammation, neuroprotection

## Abstract

Traumatic brain injury (TBI) is one of the most prevalent causes of morbidity in the United States and is associated with numerous chronic sequelae long after the point of injury. One of the most common long-term complaints in patients with TBI is sleep dysfunction. It is reported that alterations in melatonin follow TBI and may be linked with various sleep and circadian disorders directly (via cellular signaling) or indirectly (via free radicals and inflammatory signaling). Work over the past two decades has contributed to our understanding of the role of melatonin as a sleep regulator and neuroprotective anti-inflammatory agent. Although there is increasing interest in the treatment of insomnia following TBI, a lack of standardization and rigor in melatonin research has left behind a trail of non-generalizable data and ambiguous treatment recommendations. This narrative review describes the underlying biochemical properties of melatonin as they are relevant to TBI. We also discuss potential benefits and a path forward regarding the therapeutic management of TBI with melatonin treatment, including its role as a neuroprotectant, a somnogen, and a modulator of the circadian rhythm.

## 1. Introduction

Traumatic brain injury (TBI) is a prominent cause of morbidity and mortality around the world, with an estimated global incidence of nearly 1/100, as 69 million individuals sustain a TBI every year [1,2]. Patients with TBI experience a complex symptom constellation, varying widely between individuals and persisting for several years after the initial injury [3,4]. Disruption of normal sleep patterns following TBI of any severity is one of the most common complaints experienced in both acute and chronic recovery phases. Estimates for prevalence vary widely, ranging anywhere from 30% to 70% [5,6,7]. Sleep quality complaints with TBI correlate strongly with mood and pain domains. They are difficult to disentangle, as they independently associate with impaired cognition, pain sensitization, and mood disorders [8,9]; they also prolong recovery after injury [10]. Considering the high prevalence and health burden of sleep disorders following TBI, interventions that optimize sleep may provide one of the greatest opportunities to improve long-term outcomes in this population.

A suitable therapeutic intervention requires a balance of safety and efficacy, and it may be most effectively utilized with an understanding of how its mechanism of action addresses underlying injury pathophysiology. Despite growing insight into the importance of sleep dysfunction following CNS injury, detailed mechanisms for the cause of these disturbances remain largely unknown as they are likely multidimensional, depending on injury patterns and individualized comorbidities. Further, the mechanism of sleep dysfunction post-TBI may differ by chronicity since the injury. Acute parenchymal disruption has been shown to be caused by a mixture of the force-based primary injury and subsequent secondary injury, which includes resultant metabolic disruption, oxidative stress, and inflammation that may pervade long after the initial injury occurred, culminating in eventual neurodegeneration [11,12]. An ideal therapeutic might mitigate one or multiple causative injury mechanisms while simultaneously promoting a high safety and efficacy profile.

Melatonin has well-established properties as a potent antioxidant that also functions as a signaling hormone, regulating sleep and circadian physiology. For example, direct changes in melatonin production, receptor concentration, and circadian rhythm function have been repeatedly observed following TBI [13,14,15,16,17,18,19]. Disruptions of endogenous melatonin signaling after TBI may partly explain some of the pathological phenotypes related to sleep, inflammation, and hormonal function. As a therapeutic, exogenous melatonin has had several challenges. A host of negative or inconclusive clinical insomnia studies [20] conflict, in part, with supportive studies in comorbid sub-populations [21,22]. There are also some studies showing safety and efficacy in the use of melatonin for enhancing reproductive health and fertility [23,24], while some animal studies suggest there might be a risk in prenatal and childhood development due to hormone signals [25,26,27] (although no human evidence of developmental harm from melatonin is known to the authors of this paper). These data, combined with a lack of federal regulation and reports of inaccurate quantification and impurities during manufacture [28], have led to its removal from clinical guidelines and recommendations for the treatment of sleep disorders. Nonetheless, clinical use continues, and certain indications, such as the use of melatonin in shifting circadian timing (e.g., for jet lag), remain widely accepted [29] due to what appears to be an acceptable safety margin and side effect profile. Some argue that the increasingly prolific use in over-the-counter supplementation as a circadian clock-shifting stimulus and a sleep aid in the general population could be cause for concern in some susceptible populations [29].

Despite melatonin’s availability as an inexpensive, readily available “supplement”, no standard guidelines for proper dosing exist. Supplements are not regulated by the FDA, giving rise to possible drug-drug interactions and substandard quality assurance. One sample of 31 supplement manufacturers reported falsely labeled dosing by up to 400% when measured independently [28]. Although there continues to be an increasing interest in optimizing the treatment of insomnia following TBI, a lack of standardization and rigor in melatonin research has left behind a trail of non-generalizable data and ambiguous treatment recommendations. Clearly, more work is needed to investigate the true therapeutic potential of melatonin.

This review describes the underlying biochemical properties of melatonin as they are relevant to TBI. We also discuss potential benefits regarding the therapeutic management of TBI with melatonin treatment, including its role as a neuroprotectant, a somnogen, and a modulator of the circadian rhythm.

## 2. Results

Boolean searches within the PubMed database for randomized controlled trials containing the operators “TBI” AND “melatonin” returned only three results. There is a relative paucity of published works focused on the therapeutic benefit of melatonin on TBI and downstream sleep dysfunction. Expanding criteria to all article types under the same operators returned 74 results, with 40 articles found pertinent for this review. The remaining articles were collected via sampling aimed at the specific subtopics outlined in this paper (i.e., “Melatonin” AND “Physiology” OR “Anti-oxidation” OR “Neurodegeneration”) with a final total of 169 fit for inclusion. A sample of these works is presented in Table 1, Table 2 and Table 3. See Appendix A for a supplementary table (Table A1) for relevant animal model studies included for review.

## 3. Discussion

### 3.1. Traumatic Brain Injury and Sleep Disorders

Sleep disorders are pervasive following TBI. Incidence risk of insomnia, obstructive sleep apnea, circadian sleep-wake rhythm disorders, and disorders of hypersomnolence have all been shown to be increased after TBI [60]. TBI has been shown to be a risk factor associated with a higher prevalence of objective sleep disorders [61,62]. Determining the true incidence of sleep dysfunction after TBI can be difficult due to comorbid confounding, variable presentation, and incomplete pre-injury history. A review by Castriotta and Murthy found the following prevalence of sleep disorders in the TBI population: sleep apnea (23%), post-traumatic hypersomnia (11%), periodic limb movement disorder (7%), and narcolepsy (6%) [63]. Further, estimates for the prevalence of insomnia following TBI can be as high as 50% [64]. One particularly relevant variable related to the onset and severity of sleep dysfunction following TBI appears to be chronicity, whereby expressed differences are observed in acute versus chronic phases [65,66].

Acutely after injury, sleep complaints have been reported in up to one-third of patients within the first 10 days after mTBI, increasing to half of the patients within 6 weeks [67,68]. Using two- and four-week follow-up evaluations, Haboubi found the most frequent complaints following mTBI by patients to be fatigue, headache, dizziness, irritability, sleep disturbance, poor concentration, and poor memory [69]. Mathias showed that three months after TBI, 50% of individuals reported disordered sleep, and 25–29% of those were diagnosed formally with insomnia, hypersomnia, or apnea [6]. The observed incidence of sleep symptoms included 50% insomnia, 50% disordered sleep maintenance, 49% with objective poor sleep efficiency by polysomnography testing, early morning awakening at 38%, and 27% incidence of nightmares [6]. Sleep disorders may persist for years after injury, with two prospective longitudinal studies reporting that two-thirds of patients were impacted by a sleep disorder three to five years after injury [4,70].

Recently, the Transforming Research and Clinical Knowledge in Traumatic Brain Injury (TRACK-TBI) study enrolled 2022 participants to longitudinally characterize insomnia following TBI, and 61% of participants endorsed persistent insomnia up to one year following injury [71]. A separate study revealed 84% of patients with severe TBI endorsed sleep dysfunction on admission, which persisted in 66% of those one month after injury [72]. Those patients exhibiting sleep dysfunction acutely were found to have an increased risk for headaches, depression, and irritable mood.

### 3.2. Pathophysiology of TBI Associated Sleep Dysfunction

#### 3.2.1. Primary Injury

**Figure 1:**
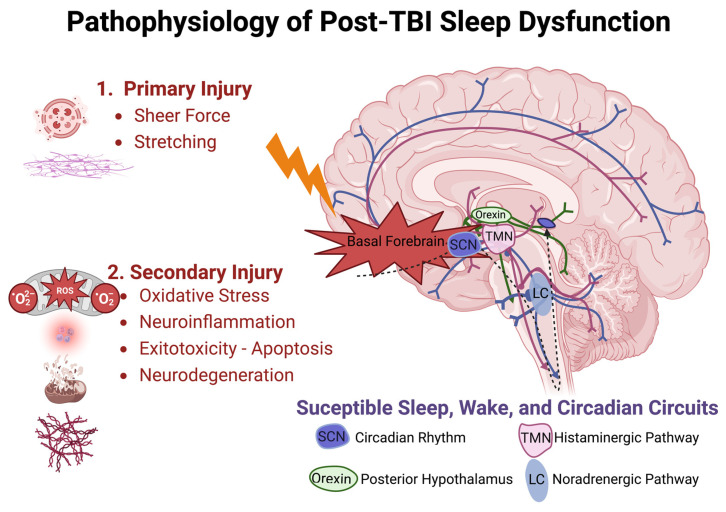
Pathophysiologic model of traumatic brain injury and disrupted sleep-related circuitry. Injury is sustained in two phases, with primary injury likely to disrupt axonal projections near the skull base and secondary injury responsible for prolonged cellular injury owed to oxidative stress, neuroinflammation, glutamate excitotoxicity, and neurodegeneration over acute and chronic durations. Suprachiasmatic Nucleus (SCN), Tuberomammillary Nucleus (TMN), Locus Coeruleus (LC).

Proposed mechanisms underlying sleep dysfunction following TBI can be subdivided by chronicity and subsequent microscopic or macroscopic effects. Acute injury mechanisms implicate acceleration-deceleration (blast and/or coup-contrecoup), resulting in axonal shearing and diffuse interruption of affiliated functional networks, theoretically including those associated with wakefulness and sleep, as shown in Figure 1 [73]. Cranial surface morphology exerts traumatic action in areas of high shear stress, such as the sphenoid ridge, inferior frontal, anterior temporal, and basal forebrain regions. These areas are rich in axonal projections mediating sleep and wakefulness, such as those from the locus coeruleus (noradrenergic pathway), the suprachiasmatic nucleus (circadian rhythm disorders), posterior hypothalamus (orexin neurons), and tuberomammillary nucleus (histaminergic pathway) [73].

Delayed mechanisms inciting injury include hypoxemia, hypotension, increased intracranial pressure, seizures, and hematoma formation. Microscopic effects of cellular damage, unchecked free radical production, neuroinflammation, and biochemical excitotoxicity-related events have all been shown to disrupt normal neural function following TBI.

#### 3.2.2. Secondary Injury

**Figure 2:**
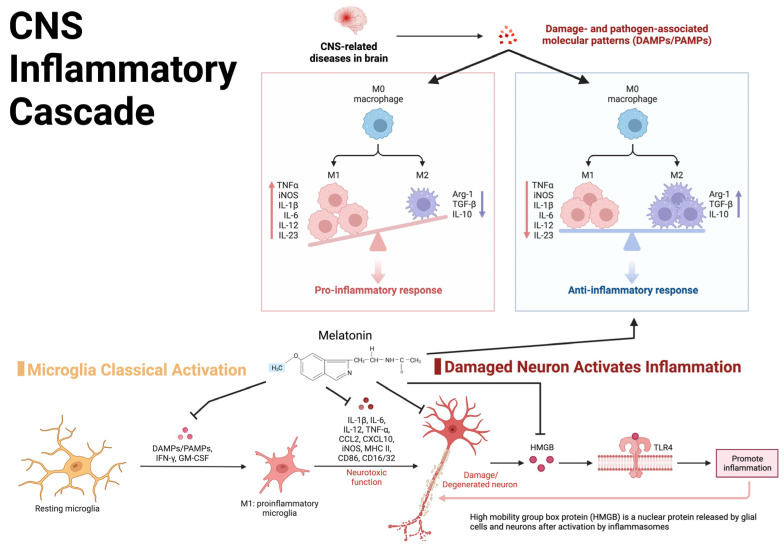
Visual representation of the central nervous system inflammatory cascade and the inflammatory feedback loop potentially leading to chronic post-TBI symptoms.

Extensive inflammatory cytokine release is observed following TBI, functioning as an innate mechanism to promote self-healing and stabilize the parenchymal microenvironment of the CNS [74,75]. However, chronic inflammation can prolong clinical recovery and predispose patients to additional deficits [36,76]. Following primary injury, mediators of inflammatory cascades are released, which in turn promote the recruitment, activation, and integration of immune cells and signaling molecules within the cerebral microenvironment, as shown in Figure 2 [75]. The inflammatory response following primary injury is a prominent catalyst for secondary insults such as ischemia, edema, hemorrhage, lipid peroxidation/free radical injury, and cell death [76,77]. Secondary injuries can prolong treatment and impair a complete, timely recovery, representing an important focus for an interventional study.

#### 3.2.3. Injury Severity

The inciting force which produces clinically recognized TBI can be as innocuous as head jostling, common to many recreational sports, or as complex as an explosion resulting in multiple pressure waves, lacerations, contusions, and fragmented bones. Severe TBI has been found to result in loss of function in wake-promoting tuberomammillary histaminergic systems and is associated with symptoms of daytime fatigue. A post-mortem examination of patients with severe TBI found a loss of 41% of histaminergic neurons, 29% melanin-concentrating hormone, and 21% of orexinergic neurons [78]. Cortical excitability and stimulation likely underpin excessive daytime sleepiness (EDS) and fatigue in the TBI population. The magnitude of the force is not predictive of the severity of the injury. The severity of a TBI is dependent on a multitude of factors, including the mechanism of injury, characteristics of the individual patient, such as age, previous central nervous system (CNS) injury, and predisposing conditions [79].

#### 3.2.4. Genetic Risk

Genomic variation may also expose individuals to the risk of developing sleep dysfunction following TBI. Genetic susceptibility for developing circadian rhythm disorders following TBI has been identified, providing additional context for mechanisms related to pathophysiology and risk factor stratification [80]. The PERIOD (Per) gene family, which is a polymorphic regulator of circadian rhythm, has been implicated in delayed sleep phase syndrome and confers increased risk for shorter sleep duration following TBI, as reported by Hong et al. [81]. Heterozygous Per3 carriers were associated with a significant risk for persistent sleep dysfunction following TBI [81]. Just as the magnitude of force does not always predict injury severity, TBI severity does not predict the severity of sleep-related symptoms; whether accounting for the genetic background would improve sleep outcome prediction remains an open question.

### 3.3. Melatonin Physiology

**Figure 3:**
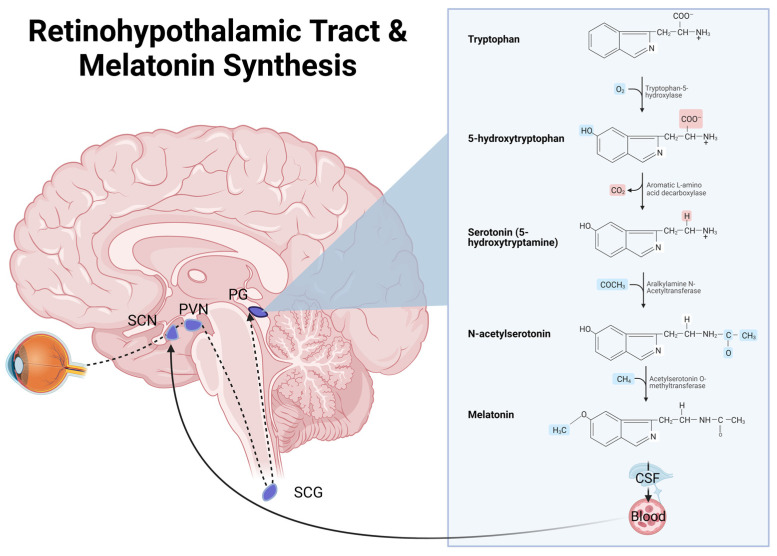
The neuroendocrine pathway as light input travels through the retina, suprachiasmatic nucleus (SCN), paraventricular nucleus (PVN), superior cervical ganglion (SCG), and pineal gland (PG).

Originally isolated from bovine pineal glands by dermatologists in 1958, melatonin was named for its ability to blanch skin cells by inhibiting melanocyte-stimulating hormone [82]. Although sleep and circadian regulation are the best-known functions of melatonin, they are evolutionarily predated by its potent antioxidant properties. The light-dependent inhibition of melatonin synthesis is a comparatively new evolutionary development, as shown in Figure 3. The pineal gland is the only endocrine organ influenced by neuronal activity (from photoperiod), primarily secreting melatonin at its highest concentrations at night. Melatonin effects and receptors have been identified in a wide range of extracerebral organs. The biodistribution of melatonin is heterogeneous; bile and CSF have much higher concentrations than plasma by several orders of magnitude.

**Figure 4:**
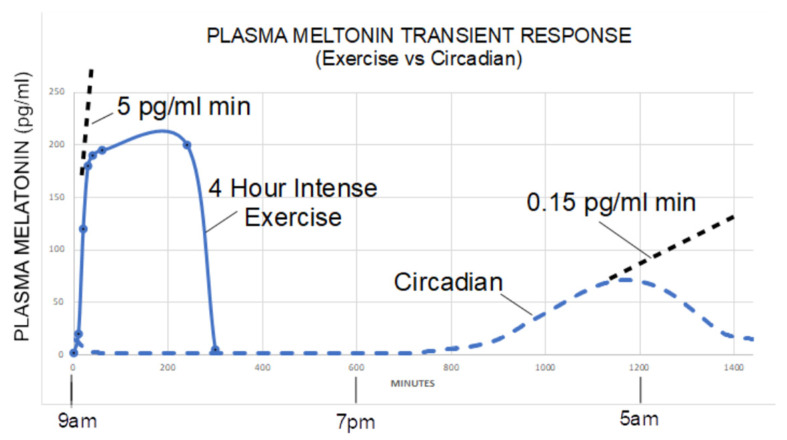
The plasma melatonin levels as a function of time during heavy exercise and circadian time. This figure was from [89] and permitted by the authors. The figure was created based on collated data from Zhu and Theros. The data set does not represent one individual but covers ranges of reported values in different light exposure settings. During a 4-h intense exercise session, plasma melatonin levels rose to 200 pg/mL in 20 min, followed by a plateau for the duration of the exercise (five test subjects with indwelling catheter measured plasma melatonin at 10, 20, 30, 40, 50, 60, 240, and 300 min, respectively).

Melatonin is synthesized in all tissues and cell types, with the majority being produced by the mitochondria [83] and to a lesser degree in the cytosol, as erythrocytes have also been shown to produce melatonin in vitro [84]. The gastrointestinal (GI) tract maintains higher levels of melatonin than the serum by 10–100 times and is more than 400 times greater than in the pineal gland, suggesting an alternative function beyond sleep regulation [85]. Melatonin cannot be stored within cells and is instead directly secreted into the cerebrospinal fluid of the third ventricle by melatonin-producing cells of the pineal gland, where it enters systemic circulation [86]. An amphiphilic molecular structure allows the molecule to diffuse across all membranes, including the blood-brain barrier, acting throughout the body and various tissue types [86], suggesting tissue-dependent function. The pool of serum and tissue-dependent concentrations appears to receive contributions from the pineal gland, dietary consumption, microbiota production, and non-visible near-infrared radiation (NIR) [87]. Interestingly, NIR penetrates inches into the human body, causing dose-dependent increases in melatonin production, likely to counteract UV damage from the sun [88]. This dose-dependent increase is by no means an insignificant or quickly resolving phenomenon either. A four-hour period of heavy exercise outdoors induces a melatonin peak approximately three times higher than the nightly circadian peak, with a rate of increase approximately 33 times faster [89]. This relationship appears to indicate a correlation between metabolically demanding processes/activities and melatonin levels, as shown in Figure 4.

Melatonin’s effects on DNA and free radical scavenging pre-date its effects on sleep by approximately 2.5 billion years, as evidenced by its production in invertebrates, plants, and unicells [90]. Melatonin is thought to have evolved in purple non-sulfur bacteria to reduce the free radical damage generated during aerobic metabolism. These bacteria were then phagocytosed by eukaryotic cells, becoming what are now mitochondria [91]. At a biochemical level, melatonin administration in humans and rats has been associated with increased levels of antioxidant enzymes, such as glutathione peroxidase and superoxide dismutase [92,93,94,95]. Some evidence suggests melatonin improves mitochondrial function via complex I/IV action in the electron transport chain by reducing acute metabolic demand [96,97,98]. Melatonin is also implicated in inhibiting programmed cell death via mitochondrial caspase/apoptosome preservation [99,100,101].

Free radical production is necessary for several physiologic processes, including energy production within mitochondria, cellular apoptosis, post-injury cytoskeletal remodeling, and normal function of the innate and adaptive immune system. Melatonin’s anti-inflammatory properties reduce detrimental neuroinflammation that impairs normal brain function [76,102,103] through cytokinetic action and through indirectly decreasing inflammatory mediators, such as nitric oxide and malondialdehyde [104]. The antioxidant effects of melatonin are more potent, per molecule, than vitamin C, vitamin E, and glutathione. A single molecule of melatonin may react with up to 10 ROSs [95], conferring benefits to human immune system regulation, tumor suppression, and neuroprotection. For example, melatonin has been shown to inhibit the expression of SIRT1, a pro-oncogenic gene product responsible for the downregulation of p53-mediated apoptosis implicated in multiple human cancers, including osteosarcoma, prostate adenocarcinoma, and retinoid orphan nuclear receptor alpha (RORα) gene-associated breast cancer [105,106]. Melatonin has also been shown to play a neuromodulatory role in TBI, exerting neuroprotective effects by reducing symptom burden following TBI [35,37]. For example, in the short term, melatonin may induce excessive glutamate release after TBI (thus inducing acute toxicity) through action at inhibitory gamma-aminobutyric acid (GABA) subset A receptors [106,107,108,109]. However, in the long term, melatonin appears to decrease neurotoxicity related to chronic traumatic encephalopathy-associated beta-amyloid aggregation [110].

Interestingly, melatonin is implicated in immune regulation and is hypothesized to be responsible for prolonging healthy aging in centenarians [111,112]. Measures of immune system function, such as T cell proliferation and cytokine production, have been shown to predict human longevity, and some evidence suggests that melatonin’s role as an antioxidant could contribute to longevity, perhaps through immune regulation [113,114]. Melatonin’s effects on electron scavenging and mitigation of inflammatory pathways, such as decreasing cytokine production, apoptosis, and circadian signaling, each have implications for understanding the pathophysiology and subsequent development of treatments for strokes, TBIs, cardiac arrest, and other organ system ischemia. 

Melatonin also exerts its effects via binding and activation of the melatonin receptors. Melatonin receptors 1A (MT1, encoded by MTNR1A) and 1B (MT2, MTNR1B), expressed in both the central nervous system and in numerous peripheral tissues, are transmembrane proteins that activate G protein-coupled receptors. Melatonin’s neuroprotective properties are mediated by its strong affinity and activation of the brain mitochondria MT1 receptor in the outer mitochondrial membrane, which inhibits the release of cytochrome c, blocking caspase activation and inhibiting apoptosis [83]. Interestingly, genetic polymorphisms have not been linked with sleep or circadian phenotypes; rather, variations of the MTNR1B gene are associated with type 2 and gestational diabetes [115,116,117,118] and also adolescent idiopathic scoliosis [119]. Other studies linked receptor polymorphisms with polycystic ovary syndrome [120] and hepatocellular carcinoma [121].

The recently discovered human glymphatic system may also be involved in and affected by melatonin. Operating as a central system of waste disposal within the CNS, disruption of normal glymphatic clearance has been implicated in increased amyloid beta and tau burden, accumulation of TBI biomarkers S100b, GFAP, NSE [122,123,124], and increased risk of chronic traumatic encephalopathy following TBI [125]. Sleep appears to be critical for normal glymphatic function, as demonstrated by Xie et al. 2013, whereby CSF delivered radio labeled tracer uptake was reduced by up to 95% in the cortex during the awake state compared to sleep in murine models [126]. During sleep, the cortical interstitium expands by up to 60%, allowing more rapid fluid clearance [126]. Melatonin is delivered by the glymphatic system to highly active parenchymal tissues, where accumulated free radicals may undergo scavenging. The dual role of melatonin as both an antioxidant and circadian hormone raises the possibility that it can influence glymphatic function, conferring a possible greater neuroprotective importance for the pineal gland by extension. The pineal gland may also be responsible for CSF secretion, a function primarily attributed to the choroid plexus [127]. The microvascular architecture of both tissues shares unique morphology with networks of the convoluted fenestrated capillary that distinctly facilitate CSF production [128]. Taken together, being responsible for melatonin synthesis and possibly CSF production may indicate a more prominent role of the pineal gland in moderating healthy glymphatic function than previously understood [129].

#### 3.3.1. TBI Effect on Melatonin Synthesis

Patients with TBI have been shown to exhibit decreased evening melatonin production by up to 42% compared to healthy controls [130]. The hypothalamic suprachiasmatic nucleus regulates the circadian rhythm, influencing various neurologic activity patterns, including the sleep-wake cycle, both directly via neurological inputs and indirectly by hormonal synthesis regulation. The best-characterized regulatory activity is stimulated by the light-activated melanopsin-expressing retinal ganglion cells, which project directly to the suprachiasmatic nucleus to activate an array of GABAergic projections [see Figure 2] to the paraventricular nucleus, which result in inhibition of melatonin synthesis in the pineal gland. This light-mediated inhibition depends upon a long, multi-synaptic pathway of sympathetic fibers that descend and synapse in the spinal cord and again at the cervical ganglia before ascending to activate melatonin synthesis in the pineal gland via beta receptor second messenger systems. In theory, long pathways may be susceptible to acceleration/deceleration forces, yet this anatomy has not been systematically examined post-TBI. Regardless of the mechanism, following TBI, melatonin levels become diminished, resulting in a higher threshold for sleep initiation and maintenance, with the most pronounced effect on sleep architecture being decreased total REM sleep. Multiple studies demonstrate altered melatonin secretion compared to controls in both acute and chronic recovery phases [31,130,131]; however, these studies are small and relatively limited to severe TBI. Structural lesions associated with fatigue include injury associated with the ascending reticular activating system, limbic system, anterior cingulate, middle frontal, and basal ganglia [132]. Further associated structures include the pontine reticular formation, posterior thalamus, midbrain processes surrounding the third ventricle, and cervical lesions involving the locus coeruleus. Whether injury to any of these structures correlates with melatonin synthesis remains untested.

Although data suggest both melatonin and its receptors decrease following TBI, the mechanism of these reductions is unclear [31,130,131]. One explanation may be that tryptophan, a melatonin precursor, is preferentially converted into kynurenine [133]. Alternatively, melatonin may be metabolized at a quicker rate following TBI due to the scavenging of free radicals. Or perhaps melatonin escapes the injured blood-brain barrier or is degraded following TBI. Further, the pineal gland itself may be sensitive to traumatic injury, impairing the body’s greatest contributor of systemic melatonin [134]. Unfortunately, the pineal gland has been largely neglected in animal models of TBI in studies analyzing melatonin and its receptors.

A previous study attributed decreases in melatonin levels to changes in melatonin metabolism [135]. Tryptophan is a naturally occurring amino acid implicated in numerous metabolic pathways. Notably, tryptophan can be metabolized into serotonin, which is further processed to create melatonin. Tryptophan may also be metabolized into kynurenine via the enzyme indoleamine 2,3, deoxygenase 1 [133]. Zhang et al. evaluated the metabolic activity of tryptophan after TBI using a pediatric rabbit model of TBI [135]. Post-mortem evaluations were performed at 6 h through 21 days post-TBI. No gender differences were noted, but researchers found indoleamine 2,3, deoxygenase 1 was upregulated at all time points in this study. Starting at 7 days post-TBI, kynurenine levels were also significantly elevated. Additionally, melatonin levels were significantly decreased at 21 days post-TBI. The authors noted a significant decrease in the melatonin/tryptophan and melatonin/serotonin ratios at 21 days post-TBI and suggested that the decrease in melatonin may be attributable to the downregulation of the melatonin pathway and upregulation of the kynurenine pathway. An alternative analysis of this study is that melatonin levels are depleted due to the binding of free radical byproducts or that melatonin is being degraded at a more rapid rate [135]. When melatonin reacts with free radicals, it may appear as a product loss on gel electrophoresis. The elevation of indoleamine 2,3, deoxygenase 1 could then be explained by an increase in tryptophan production, which is rapidly utilized to make both kynurenine and serotonin. Serotonin was unchanged between groups, suggesting changes in melatonin were likely downstream.

Considerable work has also been conducted to quantify endogenous melatonin changes after TBI in human studies [14,15,16,17,130,131,136,137]. Though most of these studies quantified melatonin in patients acutely after TBI [13,14,15,16,17,131,136], two studies investigated patients between one and six years from the initial injury [130,137]. In studies measuring melatonin in ICU patients with an acute phase TBI, five studies showed an increase in melatonin production [13,14,15,16,17], and one study showed a decrease relative to controls [130,131]. Both studies that observed patients one to six years after initial injury found lower melatonin levels relative to controls [130,137]. A large range in melatonin levels compounds the difficulty of comparing results across studies. In Seifman’s 2008 study, CSF and serum melatonin were measured, and a 5x increase in melatonin was found in the CSF of TBI patients, but no difference in serum melatonin relative to controls [13]. Subsequently, in Seifman’s 2014 study, where only serum melatonin was measured, a lower level of melatonin was found relative to healthy controls but not ICU controls [131]. These studies suggest that there are changes to melatonin levels in patients with TBI compared to the healthy population; however, the magnitude and direction of these changes are more difficult to ascertain.

Differences in the methodology of measurement, time since injury, injury severity, and patient selection may all play a role in the incongruences observed between studies. Serial measurements to normalize and align comparative measures relative to the peak or nadir of the phase response curve were not performed in most studies. In these studies, melatonin was quantified in serum [13,14,15,17,131,136], saliva [16,130,137], urine, and CSF [13]. The patient population that these samples were taken from represents a range of Glasgow Coma Scale scores from a median of 4 [17] to an average of 8.8 [137], though all studies utilized the standard definition of a severe TBI as a Glasgow Coma Scale below 8. Difficulty producing high-quality control groups among different studies is overcome in many cases with only literature-based reference value comparison [14,131,136]. This continues to be a problem as recent as 2021 [17]. In Seifman’s study, their healthy controls differed in ranges of serum melatonin concentrations from literature values. In these studies, quantified values of melatonin in TBI patients were compared against control groups that had some combination of differences in TBI severity, age, and gender [15,16,17,131]. In studies that had higher quality controls that were aged-matched, pre-existing injuries that resulted in ICU stay and uncaptured environmental factors may have also impacted melatonin production [15,16,17,131]. The lack of sampling and analytic standardization of melatonin-based clinical research led the 2005 Associated Professional Sleep Societies participants to create a working group to resolve these issues [138,139].

#### 3.3.2. TBI Effect on Melatonin Receptor Expression

Melatonin likely acts both via a receptor-independent mechanism and receptor-dependent mechanism when fulfilling its anti-inflammatory and antioxidant responsibilities [63]. Data on melatonin receptor expression change following TBI are limited in human studies. Some animal data suggest that TBI reduces melatonin receptor levels; however, the mechanism of action is unclear. In 2017, Osier et al. found that TBI, in a sample of 25 adult male Sprague Dawley rats, resulted in lower levels of the melatonin receptor subtypes MT1 and MT2 as assessed by gel electrophoresis [18]. The researchers used stereotactic neurosurgery to induce cortical damage and found that MT1 and MT2 expression was reduced in the frontal cortex at 24 h post-TBI. The hippocampus also demonstrated reduced MT1 and MT2 expression at both 6 and 24 h post-TBI, compared to controls. Actin, a popular cytoskeleton control in gel electrophoresis studies, was used as a loading control when measuring MT1 and MT2. Although actin levels were shown to be constant across experimental and control animals, the authors note that if significant gliosis and neuronal death occurred in the experimental animals, actin levels might be artificially elevated despite significant cell death; thus, leading to the false conclusion that MT1 and MT2 are less expressed. Future research is needed to further characterize exactly where MT1 and MT2 are expressed. As such, continuation in this line of research is necessary for determining whether reduced melatonin receptor expression occurs and elucidating potential links to symptoms of TBI and/or efficacy of melatonin therapy.

Other studies have sought to characterize melatonin receptor expression at remote time points. Rui et al. found that MT1 and MT2 expression is decreased for an extended period following TBI [19]. In this study, investigators sought to explore whether the deletion of Ferritin H in mice reduced melatonin’s protective nature in TBI-induced ferroptosis. Ferroptosis, a form of cellular death regulated by lipid and iron oxidation, has recently been shown to play a role in TBI. The authors found that TBI resulted in increased reactive oxygen species (ROS) production and significantly decreased MT1 and MT2 expression in the cortex at 12 h and 14 days. Interestingly, melatonin administration 1 h after TBI was sufficient to rescue MT1 and MT2 receptor levels 24 h after TBI in the wild-type group treated with melatonin; however, that effect was not observed in the Ferritin H knockout group. Melatonin and liproxstatin-1, an inhibitor of ferroptosis, were both shown to significantly decrease TBI-induced ferroptosis, lesion size, neuronal damage, and resultant behavioral deficits in the mice. The changes in MT1 and MT2 expression post-TBI are likely related to brain volume loss/neuronal damage, as melatonin was also shown to rescue their expression. The authors suggest the neuroprotective effects of melatonin are mediated by the MT1 and MT2 receptors, as their antagonists (4P-PDOT and Luzindole, respectively) appear to block melatonin’s effect, with MT2 being the major subtype involved.

### 3.4. Therapeutic Potential of Melatonin

#### 3.4.1. Melatonin’s TBI Therapeutic Potential—Circadian and Sleep-Wake Disorders

Melatonin’s potential as a therapeutic agent in treating post-TBI sleep dysfunction is owed to its ability to alter circadian rhythms and induce sleep while mediating neuroinflammation. However, due to melatonin’s classification as a supplement, the FDA does not have regulatory oversight. Despite this, various societies have recommended melatonin as an acceptable treatment in the management of primary insomnia [56,57,58,140] though no clear consensus has been reached.

Common dosing regimens for insomnia may range from 1 to 5 mg [141] but can be as low as 200 mcg and as high as 50 mg [142], typically taken a few hours prior to bedtime due to its rapid absorption and half-life between 20–50 min. Some studies report side effects, such as vivid/nightmare dreams, dizziness, daytime fatigue or hangover effect, headache, depression, irritability, and stomach cramps [143]. However, a recent review that included 5 RCTs did not show any serious adverse effects in adult or adolescent populations [42].

Evidence for melatonin’s effects in TBI and non-TBI populations is mixed. In the non-TBI population, melatonin supplementation provides a small benefit for sleep onset but none for maintenance [36,144]. These results are confounded by differences in dose, time since injury, and individual physiological differences, all of which impact effect size. In 2019, Barlow et al. identified 12 meta-analyses of placebo-controlled randomized trials (3–13/study) with varying degrees of methodologic quality, finding a statistically significant (though low in magnitude) improvement in sleep latency and total sleep time [36]. Uncertainty remains whether these small changes are clinically significant.

One RCT associated with the PLAYGAME trial enrolled 99 participants aged 8–18 years old with mTBI and post-concussive symptoms into either a treatment group consisting of 3 mg or 10 mg of melatonin compared to the control. No symptom change was observed [47]. However, melatonin has been shown to be effective at treating children with TBI-associated insomnia [145] and was also independently shown to exert neuroprotective effects in the neonatal and pediatric TBI populations [36].

One randomized controlled trial in adults comparing melatonin to amitriptyline found melatonin improved daytime alertness compared to controls, but no alterations in sleep latency or duration were observed [34,146]. Additionally, one RCT found a 4-week supplementation regimen to be safe and effective for improving sleep quality in patients with prior TBI [44]. Another RCT demonstrated melatonin replacement may be effective in resetting sleep-wake cycling with an observed positive impact on daytime fatigue [43]. Melatonin was found to modulate sleep and wake rhythm and subsequently was beneficial for post-TBI sleep disorders [40].

Ramelteon (a melatonin receptor agonist) is approved by the FDA for insomnia with sleep onset dysfunction, exerting its action by decreasing evening SCN-driven arousal, which helps reinforce circadian periodicity [147,148]. Its use is associated with low risk due to its favorably low side effect profile, with reported limited adverse effects on neurobehavioral function. Ramelteon has been shown to improve PSQI scores for patients with mild to severe TBI in addition to an associated increase in total sleep time and variable cognitive functioning following a 3-week trial at 8 mg compared to [45]. However, ramelteon was not associated with improved sleep onset latency in TBI populations despite an overall improvement in sleep quality.

In one systematic review, methylphenidate and melatonin were the only pharmacological interventions shown to reduce fatigue in patients who suffered from post-traumatic brain injury fatigue (PTBIF) [39]. Interestingly, ramelteon did not demonstrate this effect. The effect of melatonin on reducing PTBIF may be attributed secondarily to improved sleep quality and might only improve sleep-related fatigue [39].

The therapeutic potential of melatonin to address sleep-wake disorders after TBI is promising. Further work is needed (such as FDA regulation) to improve the reliability of the dosing. This, in combination with a detailed understanding of pharmacokinetics, will facilitate the development of biomarkers of target engagement and pharmacodynamic effect. By establishing these basic parameters, the promising data noted in previous paragraphs can be validated in rigorous follow-on clinical trials.

#### 3.4.2. Melatonin’s TBI Therapeutic Potential—Antioxidant/Anti-Inflammatory

Melatonin administration after TBI may confer benefit through its potent anti-inflammatory and anti-oxidative properties. CNS injury frequently results in wide cellular process disruption, often involving metabolic cascades, mass neurotransmitter release, mass free radical release, increased oxidative stress, and mitochondrial dysfunction. Melatonin contains an electron-rich aromatic ring that enables melatonin to act as an electron donor and subsequently reduce the concentration of free radicals during periods of oxidative stress [76]. Oxidative stress develops when oxygen byproducts from the electron transport chain contain unstable electrons that then react to form ROS beyond the levels of antioxidants, thereby increasing the concentrations of free radicals [149]. At supra-physiologic levels, as seen with supplementation, melatonin can exert both receptor-mediated and intracellular effects, capable of diffusing to the microenvironment of cellular injury to perform its oxidative scavenging function [76,111,150,151,152,153,154,155]. Free radical production, while necessary for energy production in mitochondria, apoptosis, clearing of post-injury biological debris, and immune defenses, is a double-edged sword. The risks of normal free radical production are mitigated by antioxidants, such as melatonin, glutathione, and vitamins E and C, that deactivate ROS responsible for collateral damage during normal function.

The anti-inflammatory actions of melatonin are accomplished via indirect and direct means, most of which occur without the help of a known receptor. However, melatonin is reported to bind to quinone reductase 2, which serves as an indirect antioxidant that enhances the abilities of other antioxidant enzymes [76]. Another highlighted mechanism includes the activation of nuclear factor-erythroid 2 related factor 2, antioxidant-response element (Nrf2-ARE), and increased downstream factors [156]. It was found that melatonin administration in post-TBI mice limited neuronal degeneration near lesions, edema, and levels of oxidative products, such as malondialdehyde (MDA), a product of oxidative stress, and 3-nitrotyrosine (3-NT), a marker of nitrogen free radical species. It was also found that melatonin administration returned levels of antioxidant enzymes to normal compared to the non-melatonin control groups. Interestingly, melatonin also influences the way macrophages and microglia respond, driving differentiation into the anti-inflammatory M2 type [157].

The timing and route of melatonin administration can play a critical role in the physiologic response to injury and treatment. Findings from a study on the impact of intracoronary and intravenous melatonin administration in patients receiving percutaneous coronary intervention following a first-time ST-elevation myocardial infarction (STEMI) showed evidence of a potential golden period in which free radical damage is preventable and even reversible before cells undergo irreparable damage [50]. Treatment was given both before and after the restoration of blood flow to the infarcted vessel. Researchers found that melatonin was associated with a significant reduction in infarct size when given early after symptom onset [50]. A follow-up pilot RCT showed increased survival rates among melatonin recipients as measured by mortality and heart failure readmission at two years [51]. Interestingly, in the short term, there was no improvement in myocardial salvage index on cardiac MRI at day 4 (+/−1) after intravenous melatonin administration following STEMI. This may suggest the bulk of melatonin’s benefits are more evident in the long term, potentially via enhanced repair mechanisms as opposed to damage prevention [52].

The effects of melatonin may be blunted via oral administration. Patients undergoing coronary artery bypass graft surgery (CABG) experienced no antioxidant benefit from oral melatonin; however, there was a significant increase in ejection fraction and a decrease in heart rate with melatonin [53]. Intraperitoneal melatonin administration in rats after a surgically induced myocardial infarction (MI) was associated with an increased plasma level of *Sirt6*, a stress response protein involved in metabolic pathways affecting DNA repair, ATP production, and inflammation. Evidence shows melatonin may significantly increases *Sirt6* mRNA transcription while reducing the levels of iNOS and phosphorylated iNOS after ischemic insult [158,159]. While one study found decreased myocardial infarct size and ROS levels following melatonin administration, these findings were not statistically significant [159].

Evidence for the benefit of intraperitoneal melatonin benefit in animal models is encouragingly more robust in the context of hemorrhagic and ischemic brain injury. Much like TBIs, ischemic strokes promote an inflammatory response that perpetuates the production of cytokines and oxidative stress through the initiation of various inflammatory cascades [160]. Intraperitoneal melatonin administration has been associated with attenuated secondary brain injury after intracerebral hemorrhage in rat models [161]. Benefits included significantly reduced blood-brain barrier (BBB) disruption, decreased indicators of oxidative stress, inflammation, and DNA damage, reduced pro-inflammatory cytokines, increased antioxidant protein levels, reduced infarct size, improved sensorimotor functional deficits at early time points, and reduced percentage of apoptotic cells in a dose-dependent manner [160,161].

The success of melatonin as an antioxidant treatment for ischemic strokes has also been shown in humans, as evidenced by positive outcomes in newborns with hypoxic-ischemic encephalopathy (HIE) after cerebral hypoxia during birth. Such insults are associated with delayed development or premature death. Newborns have a decreased production of melatonin until months after their birth [162]. Researchers used melatonin as an adjuvant therapy to the standard of care for mild hypothermia and found improved outcomes as well as reduced inflammation and oxidative stress [151,162,163,164]. The decreased melatonin production in newborns may be offset by different pharmacokinetic activity than their adult counterparts. Melatonin has been shown to have a longer half-life in newborns compared to adults, which may explain the decrease in endogenous melatonin production [130,165]. Whether these data have implications for the therapeutic use of melatonin as an antioxidant after TBI remains unclear. Future studies in TBI patients should account for endogenous levels of melatonin over multiple time points in addition to assessment of melatonin receptor genetic haplotypes and expression when possible.

Most TBI treatments are largely aimed at symptom management until self-resolution [166]. Previous TBI treatment trials involved progesterone [167], magnesium (Mg) [168], erythropoietin (EPO) [169,170], hyperbaric oxygen therapy (HBOT) [171], cyclosporin [172,173], and IV corticosteroids [173]. Cochrane reviews of Mg and HBOT did not show evidence to support treatment, although HBOT may cautiously be interpreted to have a decreased mortality rate [168,171]. Except for IV corticosteroids, EPO, cyclosporine, and progesterone showed no difference compared to placebo in terms of safety and efficacy. Interestingly, IV corticosteroids were associated with an increased mortality rate within the first two weeks [173]. In contrast, melatonin’s safety and positive effect has been demonstrated in neonates [162,164], children/adolescents [143], and adults via multiple routes of administration, including oral, IV, and intracoronary routes [50,52,174,175]. Doses as high as 300 mg via PO without significant clinical effects have been demonstrated [176]. An RCT performed in ICU patients showed median serum melatonin levels of 150 pg/mL (range, 125–2125 pg/mL) with an oral dose of 10 mg [177]. Considering physiologic exercise-induced melatonin increases to over 200 pg/mL have been demonstrated [89], it is likely that a large portion of the healthy population have experienced similar levels at multiple times in their lives.

## 4. Materials and Methods

A comprehensive, nonsystematic, narrative review utilizing standardized search strategies was conducted within the PubMed database. Various Boolean inputs were combined; an inexhaustive list includes the following terms: Melatonin, Safety, Efficacy, Neurodegeneration, Neuroinflammation, TBI, Concussion, Head Trauma, Insomnia, Human, Animal Model, and RCT. Included review study designs consist of meta-analyses, systematic reviews, literature reviews, and book chapters, while experimental designs included randomized controlled trials and animal studies, which contributed to the understanding of the biochemical and physiologic mechanisms of melatonin changes after injury, the role of melatonin in neuro-inflammatory mediation and sleep regulation and therapeutic potential of melatonin following TBI. Additional articles were selected based on the references contained in the articles. Inclusion was determined by meeting one or more of the following: (a) animal TBI models investigating the therapeutic mechanism of melatonin, (b) human subjects with sleep disruption following TBI, (c) melatonin characterized as therapeutic, (d) pathophysiologic investigation of sleep disruption following TBI in human subjects, and (e) papers describing measurements of melatonin in healthy humans, TBI patients, and animal models were included, including reviews where primary source data were verified.

For purposes of injury stratification, TBI of all severity (mild, moderate, and severe) in addition to post-concussive syndrome were included. Further inclusion focusing on the anti-inflammatory and anti-oxidative properties of melatonin resulted in additional RCT efficacy human trials for both non-traumatic brain injury and non-CNS effects across multiple systems. Six independent reviewers assessed articles for eligibility and pertinence.

## 5. Conclusions

This review aimed to investigate recent literature to characterize the role of endogenous melatonin in TBI physiology and the use of exogenous melatonin as a treatment for TBI. A volume of pre-clinical studies consisting primarily of murine models has characterized the potential pathophysiologic mechanisms of post-TBI sleep dysfunction, including melatonin synthesis, variable receptor expressivity, and cellular susceptibility to inflammatory and oxidative damage. Pre-clinical studies regarding the treatment efficacy of melatonin in murine TBI models in treating sleep dysfunction demonstrated overall positive effects on the improvement in post-TBI behavioral, sleep, and motor outcomes. Multiple human RCTs show promising results in sleep and mental health outcomes, with evidence supporting melatonin’s utility as an antioxidant and as a signaling molecule. Future studies will need to utilize better standardization of melatonin measurements and therapeutic supply, assess longitudinal exposure, and expand to multiple sub-populations. In general, the benefits of melatonin largely appear to outweigh the harms, and the current body of evidence supports a more-disciplined look at its promise as a therapeutic to mitigate not only sleep and circadian disorders but also inflammatory sequelae in the TBI population.

## Figures and Tables

**Table 1:** Previous reviews on TBI, sleep dysfunction, and melatonin.

Citation	Title	Design	Key Findings
Kaleyias and Kothare, 2022 [30]	Sleep Disorders in Traumatic Brain Injury	Literature Review	Factors implicated in sleep disturbance following TBI include reduced hypocretin signaling, damage to histaminergic tuberomammillary neurons, disruption of circadian regulation impairing melatonin synthesis, and parenchymal damage involving the ascending reticular activating system, basal ganglia, and limbic system. Human observational studies implicate substantial loss of histaminergic neurons and impaired melatonin synthesis as significant pathophysiologic contributors up to 6 months after injury.
Naseem and Parvez, 2014 [31]	Role of Melatonin in Traumatic Brain Injury and Spinal Cord Injury: A Review	Literature Review 9 Studies	In animal models, melatonin has neuroprotective effects on both TBI and spinal cord injury (SCI). Mechanisms for observed benefit are largely owed to anti-inflammatory and anti-oxidative action leading to a reduction in cerebral edema, decreased NFkB, decreased AP-1, stabilization of Nitric Oxide Species (NOS), increased superoxide dismutase and glutathione peroxidase. Measurements of melatonin in the CSF increase acutely following TBI.
Stewart et al., 2022 [32]	Treating Sleep Disorders Following Traumatic Brain Injury in Adults: Time for Renewed Effort?	Systematic Review 18 Articles	Pathophysiology of sleep disruption following TBI remains poorly understood. Circadian rhythm dysfunction was common acutely (10 days), and low melatonin production is found up to a year post injury and associated with reduced sleep quality. Recommendation for clinical use of melatonin to treat sleep dysfunction following TBI is supported but cautioned against given the paucity of published data from human RCTs.
Gagner et al., 2015 [33]	Sleep-wake Disturbances and Fatigue after Pediatric Traumatic Brain Injury: A Systematic Review of the Literature.	Systematic Review	From over 20 identified pathologic characteristics from human and animal models from studies investigating neuropathology, only 4 were observed concurrently in both. Shared findings included decreased hypothalamic orexin, increased slow waveform during wakefulness on EEG, increased sleep fragmentation, and increased sleep time, suggesting higher validity and utility for these findings when investigating the pathophysiologic mechanism for sleep dysfunction after TBI.
Driver and Stork, 2018 [34]	Pharmacological Management of Sleep After Traumatic Brain Injury	Literature Review	Melatonin administration following TBI may improve subjective daytime alertness, but a comprehensive understanding of its restorative impact on sleep fragmentation is limited by a lack of rigorous RCTs with objective sleep data. In one double-blind placebo-controlled trial of 13 individuals with TBI, the melatonin agonist Ramelteon improved total sleep duration and cognitive performance following the 3-week trial.
Osier et al., 2018 [35]	Melatonin as a Therapy for Traumatic Brain Injury: A Review of Published Evidence	Literature Review 22 articles	In animal models, melatonin conferred neuroprotective benefits following TBI via antioxidative action, downregulation of NFkB and AP-1, and decreased apoptosis leading to reduced contusion volume during the evening. Majority of reports support the potential use of melatonin in treating human patients following TBI.
Barlow et al., 2019 [36]	Melatonin as a Treatment after Traumatic Brain Injury: A Systematic Review and Meta-Analysis of the Pre-Clinical and Clinical Literature	Meta-analysis 17 studies	From 15 pre-clinical studies, melatonin had an overall beneficial effect on subject outcomes with improvement in cognitive performance and motor function. Pertinent clinical trials included a post-concussive pediatric population that benefited from melatonin supplementation to reduce post-traumatic headaches (*N* = 12).
Blum et al., 2021 [37]	Melatonin in Traumatic Brain Injury and Cognition	Literature Review 11 studies	Murine models continue to demonstrate melatonin exerting potent neuroprotective action via anti-inflammatory and antioxidant functions. Evidence for reduced expression of abnormal proteins, including AB and p-tau, following treatment with melatonin after injury highlight a potential future application in decreasing the risk of neurodegenerative disease for which TBI exposure is a risk factor. Longitudinal data on cognitive performance in a treatment population are lacking; however, some evidence for improvement in memory task function acutely after injury does exist.
Feinberg et al., 2021 [38]	Association of Pharmacological Interventions with Symptom Burden Reduction in Patients with Mild Traumatic Brain Injury: A Systematic Review	Systematic Review 23 studies	Review of 23 studies (11 randomized clinical trials, 7 prospective observational studies, 3 retrospective observational studies, and 2 case studies) examining 20 pharmacological interventions; while methylphenidate, sertraline hydrochloride, ondansetron, amitriptyline, and melatonin were adequately represented— consistent symptom burden reduction was limited.
Ali et al., 2022 [39]	Fatigue After Traumatic Brain Injury: A Systematic Review	Systematic Review	Review of 37 articles showed methylphenidate and melatonin were the only pharmacological agents associated with decreased fatigue in RCTs.
Samantaray et al., 2009 [40]	Therapeutic Potential of Melatonin in Traumatic Central Nervous System Injury	Mini Review	Mini review exploring and summarizing characteristics and benefits of melatonin as neuroprotectant/treatment for acute SCI or traumatic CNS injuries.
Reiter et al., 2016 [41]	Melatonin as an Antioxidant: Under Promises but Over Delivers	Literature Review	Review articles summarizing the evolutionary history of melatonin as well as its biochemical pathways and physiological effects in healthy and injured states.
Cassimatis et al., 2022 [42]	The Utility of Melatonin for the Treatment of Sleep Disturbance Following Traumatic Brain Injury	Literature Review 9 studies	A total of 5 RCTs on adults and adolescents showed that post-TBI melatonin treatment improved subjective and objective sleep measures as well as mental health symptoms, executive function, and cognition.

**Table 2:** Previous human studies on TBI, sleep dysfunction, and melatonin.

Citation	Title	Design	Key Findings
Kemp et al., 2004 [43]	The Value of Melatonin for Sleep Disorders Occurring Post-Head Injury: a Pilot RCT	1 mth, Double blind crossover (*N* = 7) of TBI patients with insomnia; melatonin 5 mg/d vs. amitriptyline 25 mg/d; 2 wk washout	Melatonin improved daytime alertness compared to baseline (*d* = 0.42). No treatment effect on insomnia (F (2.48) = 0.98, *p* > 0.056) was found.
Grima et al., 2018 [44]	Efficacy of Melatonin for Sleep Disturbance Following Traumatic Brain Injury: A Randomized Controlled Trial	4 wk, Double blind crossover (*N* = 33) of TBI patients with chronic insomnia; melatonin 2 mg/d vs. placebo; 48 h washout	Melatonin improved sleep quality compared to placebo by PSQI (*d* = 0.46; *p* < 0.0001). Melatonin improved sleep efficiency (*d* = 0.28, *p* = 0.04) but had no effect on sleep onset latency (*d* = 0.18; *p* = 0.23). No treatment effect on daytime sleepiness by ESS (*d* = 0.17, *p* = 0.15)
Lequerica et al., 2015 [45]	Pilot Study on the Effect of Ramelteon on Sleep Disturbance After Traumatic Brain Injury: Preliminary Evidence from a Clinical Trial	3 wk, Double blind crossover (*N* = 13) of TBI patients with circadian rhythm disorder	Ramelteon 8 mg nightly improved total sleep time and slightly increased sleep latency. Improvement seen from psychometric tests in executive function.
Ilyer et al., 2020 [46]	Neural Correlates of Sleep Recovery following melatonin Treatment for Pediatric Concussion: A Randomized Controlled Trial	Double-blind RCT of pediatric cohort with post-concussion symptoms (*N* = 62). 3 mg vs. 10 mg melatonin vs. placebo.	fMRI findings show increased connectivity of posterior default mode networks in the melatonin group.
Barlow et al., 2020 [47]	Efficacy of Melatonin in Children with Postconcussive Symptoms: A Randomized Clinical Trial	Double-blind RCT of 99 adolescents with PPCS. Placebo vs. 3 mg vs. 10 mg.	No significant difference in outcomes on Post-Concussion Symptom Inventory score measured after 28 days of treatment. However, caveated by wide confidence intervals.
Kuczynski et al., 2013 [48]	Characteristics of Post-traumatic Headaches in Children Following Mild Traumatic Brain Injury and their Response to Treatment: A Prospective Cohort.	Prospective pediatric cohort with post-mTBI symptoms (*N* = 670; 385 males, 285 females) and comparison group with extracranial injury (*N* = 120; 61 males, 59 females). Retrospective chart review of a separate cohort (treatment cohort) treated for post-traumatic headaches (PTH) with amitriptyline, flunarizine, topiramate, and melatonin, (*N* = 44; 29 females, 15 males).	Headaches in 9/12 (75%). 13/18 patients (68%) reported a good effect with amitriptyline.
Grima et al., 2021 [49]	Poorer Sleep Quality Predicts Melatonin Response in Patients with Traumatic Brain Injury: Findings from a Randomized Controlled Trial	Secondary analysis of phase 3 randomized, placebo-controlled, double-blind, 2-period, 2-treatment crossover clinical trial evaluating the efficacy of melatonin (2 mg, prolonged release) treatment for sleep disturbances in patients with TBI	Severe TBI patients with comorbid insomnia and poorer sleep quality experience most benefit regardless of time since injury, demographics, fatigue, daytimes sleepiness, mood, and anxiety.
Dominguez-Rodriguez et al., 2017 [50]	Usefulness of Early Treatment with Melatonin to Reduce Infarct Size in Patients With ST-Segment Elevation Myocardial Infarction Receiving Percutaneous Coronary Intervention (From the Melatonin Adjunct in the Acute Myocardial Infarction Treated With Angioplasty Trial)	Multi-site, double-blind, RCT of STEMI patients in 3 groups. Placebo vs. intracoronary melatonin vs. intravenous melatonin.	Melatonin treatment in STEMI patients who present early after symptom onset was associated with a significant reduction in the infarct size after pPCI.
Dominguez-Rodriguez et al., 2022 [51]	Early Treatment of Acute Myocardial Infarction with Melatonin: Effects on MMP-9 and Adverse Cardiac Events	Pilot RCT of melatonin treatment vs. placebo in acute MI patients receiving percutaneous intervention (*N* = 94).	Melatonin associated with improved outcomes in acute MI patients undergoing primary percutaneous intervention.
Ekeloef et al., 2017 [52]	Effect of Intracoronary and Intravenous Melatonin on Myocardial Salvage Index in Patients with ST-Elevation Myocardial Infarction: A Randomized Placebo Controlled Trial.	RCT of STEMI patients in 3 groups. Placebo vs. intracoronary melatonin vs. intravenous melatonin.	No improvement in myocardial salvage index after primary percutaneous coronary intervention in patients with STEMI treated with melatonin vs. placebo.
Dwaich et al., 2016 [53]	Melatonin Effects on Myocardial ischemia-reperfusion Injury: Impact on the Outcome in Patients Undergoing Coronary Artery Bypass Grafting Surgery	RCT of 45 patients split into 3 groups: Placebo-controlled group, low dose melatonin group, 10 mg capsule once daily and high dose melatonin group 20 mg capsule once daily.	Dose-dependent melatonin supplementation can ameliorate the degree of myocardial ischemic-reperfusion injury.

**Table 3:** Previous Recommendations for Melatonin Supplementation in Sleep Disorders.

Indication	Administration	Recommendations
ASWPD		AASM Consensus panel did not provide a recommendation regarding the use of melatonin for ASWPD [54]
DSWPD	Adult: “Strategically timed,” administration: 0.3–3.0 mg; 1.5–6.5 h prior to DLMO, i.e., 15:00–21:30 for most adult patients Children (6–12 years): 1.5–2.0 h prior to usual sleep time for patients with no comorbidities, with depression, patients without depression; 20–30 min prior to caregivers’ desired bedtime or 18:00, 19:00, for those with comorbid psychiatric disease	AASM Consensus panel, rating in favor of the use of melatonin, as opposed to no treatment. [WEAK] [54]
N24SWPD	For “blind adults:” 0.5–10 mg one hour prior to desired bedtime, or consistently at 21:00	Consensus panel, rating in favor of the use of melatonin, as opposed to no treatment. [WEAK] [54] No recommendation made for sighted adults with N24SWD.
Shift work disorder	1.8–3.0 mg prior to the desired sleep period	Administration of melatonin prior to daytime sleep is indicated to promote daytime sleep among night shift workers. [GUIDELINE] [55] Melatonin improved daytime sleep but did not improve nighttime alertness (work shift alertness).
Jetlag	0.5–10 mg administered at bedtime	Melatonin administered at the appropriate time is indicated to reduce symptoms of jet lag and improve sleep following travel across multiple time zones. [STANDARD] [55]
Insomnia	Doses ranging from 0.5–10 mg have been studied for insomnia, but evidence-based guidelines were based on studies using 2 mg	Most evidence-based guidelines recommend against the use of melatonin for insomnia (compared to no treatment), based on low quality evidence, with a limited dose range, failing to demonstrate efficacy). [WEAK] [56,57,58]
TBI: misc. sleep dysfunction	3–10 mg in pediatric and adult patients	Conflicting results, increased daytime alertness, no significant impact on sleep measures [43,59].

Abbreviations: Advanced Sleep Wake Phase Disorder (ASWPD), Delayed Sleep Wake Phase Disorder (DSWPD), Non-24 Sleep Wake Phase Disorder (N24SWPD), Irregular Sleep Wake Rhythm Disorder (ISWRD), Dim Light Melatonin Onset (DLMO).

## Data Availability

Not applicable.

## Appendix A

**Table A1:** Previous animal model studies on TBI, sleep dysfunction, and melatonin.

Citation	Design	Title
Rehman et al., 2019 [178]	RCT Mice	Neurological Enhancement Effects of Melatonin Against Brain Injury-Induced Oxidative Stress, Neuroinflammation, and Neurodegeneration via Ampk/Creb Signaling
Naeser et al., 2016 [179]	LED Human	Transcranial, Red/Near-Infrared Light-Emitting Diode Therapy to Improve Cognition in Chronic Traumatic Brain Injury
Ge et al., 2020 [180]	Rats	Effect of Melatonin on Regeneration of Cortical Neurons in Rats with Traumatic Brain Injury
Ozdemir et al., 2005 [181]	Rats	Protective Effect of Melatonin Against Head Trauma-Induced Hippocampal Damage and Spatial Memory Deficits in Immature Rats
Bao et al., 2019 [182]	Rats	Silencing of A20 Aggravates Neuronal Death and Inflammation After Traumatic Brain Injury: A Potential Trigger of Necroptosis
Osier et al., 2017 [18]	RCT Rats	Brain Injury Results in Lower Levels of Melatonin Receptors’ Subtypes MT1 and MT2
Wang et al., 2012 [183]	Model	Melatonin Activates the NRF2-Are Pathway When It Protects Against Early Brain Injury in a Subarachnoid Hemorrhage Model
Ding et al., 2014 [156]	Model	Melatonin Stimulates Antioxidant Enzymes and Reduces Oxidative Stress in Experimental Traumatic Brain Injury: The NRF2-Are Signaling Pathway as a Potential Mechanism
Senol et al., 2014 [184]	Rats	Melatonin Reduces Traumatic Brain Injury-Induced Oxidative Stress in the Cerebral Cortex and Blood of Rats
Ding et al., 2015 [185]	Rats	Melatonin Protects the Brain from Apoptosis by Enhancement of Autophagy After Traumatic Brain Injury in Mice
Ates et al., 2006 [186]	Rats	Effect of Pinealectomy and Melatonin Replacement on Morphological and Biochemical Recovery After Traumatic Brain Injury
Beni et al., 2004 [187]	Rats	Melatonin-Induced Neuroprotection after Closed Head Injury is Associated with Increased Brain Antioxidants and Attenuated Late-Phase Activation of Nf-кB and Ap-1
Campolo et al., 2013 [102]	Rats	Combination Therapy with Melatonin and Dexamethasone in a Mouse Model of Traumatic Brain Injury
Dehghan et al., 2013 [188]	Rats	Effect of Melatonin on Intracranial Pressure and Brain Edema Following Traumatic Brain Injury: Role of Oxidative Stresses
Ding et al., 2014 [189]	Rats	Melatonin Reduced Microglial Activation and Alleviated Neuroinflammation Induced Neuron Degeneration in Experimental Traumatic Brain Injury: Possible Involvement of Mtor Pathway
Ding et al., 2015 [185]	Rats	Melatonin Protects the Brain from Apoptosis by Enhancement of Autophagy After Traumatic Brain Injury in Mice
Kabadi et al., 2010 [190]	Rats	Posttreatment With Uridine and Melatonin Following Traumatic Brain Injury Reduces Edema in Various Brain Regions in Rats
Kelso et al., 2011 [191]	Rats	Melatonin and Minocycline for Combinatorial Therapy to Improve Functional and Histopathological Deficits Following Traumatic Brain Injury
Lin et al., 2016 [192]	Rats	Melatonin Attenuates Traumatic Brain Injury-Induced Inflammation: A Possible Role for Mitophagy
Mesenge et al., 1998 [193]	Rats	Protective Effect of Melatonin in a Model of Traumatic Brain Injury in Mice
Sarrafzadeh et al., 2000 [194]	Rats	Neuroprotective Effect of Melatonin on Cortical Impact Injury in the Rat
Wu et al., 2016 [195]	Rats	Melatonin Attenuates Neuronal Apoptosis Through Up-Regulation of K(+)-Cl(−) Cotransporter KCC2 Expression Following Traumatic Brain Injury in Rats
Yamakawa et al., 2017 [196]	Rats	Manipulating Cognitive Reserve: Pre-injury Environmental Conditions Influence the Severity of Concussion Symptomology, Gene Expression, and Response to Melatonin Treatment in Rats
Ran et al., 2021 [160]	Rats	Melatonin Protects Against Ischemic Brain Injury by Modulating pi3K/Akt Signaling Pathway via Suppression of Pten
Wang et al., 2022 [159]	Rats	Melatonin Protected Against Myocardial Infarction Injury in Rats Through a SIRT6-Dependent Antioxidant Pathway
Rui et al., 2021 [19]	Mice	Deletion of Ferritin H in Neurons Counteracts the Protective Effect of Melatonin Against Traumatic Brain Injury-induced Ferroptosis
